# Imaging of reactive oxygen species in focal ischemic mouse brain using a radical trapping tracer [^3^H]hydromethidine

**DOI:** 10.1186/s13550-015-0115-1

**Published:** 2015-06-26

**Authors:** Kohji Abe, Misato Tonomura, Miwa Ito, Nozomi Takai, Natsumi Imamoto, Takemi Rokugawa, Sotaro Momosaki, Kazumi Fukumoto, Kenji Morimoto, Osamu Inoue

**Affiliations:** Department of Drug Metabolism & Pharmacokinetics, Research Laboratory for Development, Shionogi & Co., Ltd., 3-1-1 Futaba-cho, Toyonaka, Osaka 561-0825 Japan; Department of Applied Chemistry & Analysis, Research Laboratory for Development, Shionogi & Co., Ltd., 3-1-1 Futaba-cho, Toyonaka, Osaka 561-0825 Japan; Graduate School of Medicine, Osaka University, 1-7, Yamadaoka, Suita, Osaka 565-0871 Japan; Hanwa Intelligent Medical Center, Hanwa Daini Senboku Hospital, 3176, Fukaikita-machi, Nakaku, Sakai, Osaka 599-8271 Japan

**Keywords:** Cerebral ischemia, Middle cerebral artery occlusion, Reactive oxygen species

## Abstract

**Background:**

Reactive oxygen species (ROS) have been implicated in the pathophysiology of the brain after ischemic stroke. In this study, we investigate the generation of brain ROS after transient focal ischemia in mice using a radical trapping radiotracer, [^3^H]-labeled *N*-methyl-2,3-diamino-6-phenyl-dihydrophenanthridine ([^3^H]hydromethidine), which we recently reported as a ROS imaging probe. We also examined the effect of dimethylthiourea (DMTU), a hydroxyl radical scavenger, on brain ROS generation and infarct volume after transient focal ischemia in mice.

**Methods:**

[^3^H]Hydromethidine was intravenously injected into mice at 1, 2, 5, and 7 h after transient middle cerebral artery occlusion (tMCAO), and then, the brain autoradiogram was acquired at 60 min after tracer injection. Brain infarct volumes at 24 h after tMCAO were assessed by 2,3,5-triphenyltetrazolium chloride staining.

**Results:**

Accumulation of radioactivity was observed in the ipsilateral striatum and cortex at 1 h after tMCAO. The increase of radioactivity was attenuated at 2 h after tMCAO and then became maximized at 5 h. The high accumulation of radioactivity remained until 7 h after tMCAO. DMTU treatment significantly attenuated the accumulation of radioactivity in the ipsilateral hemisphere at 1, 5, and 7 h after tMCAO. Brain infarct volumes were also significantly reduced in DMTU-treated mice at 24 h after tMCAO.

**Conclusions:**

These results indicated that [^3^H]hydromethidine is a useful radiotracer for detecting in vivo brain ROS generation such as hydroxyl radical after ischemic injury.

## Background

Reactive oxygen species (ROS) have been implicated in the pathogenesis of various neurological disorders, such as ischemia, trauma, and degenerative disease [1–3]. Ischemic stroke can induce pathophysiological events such as amino acid excitotoxicity, ionic imbalance, and oxidative stress [4, 5]. Cumulative evidence suggests that ROS are important mediators of cell injury in the ischemic brain [6, 7]. Oxidative stress can primarily lead to the formation of superoxide radical (O_2_ · −) and nitric oxide (·NO), and then, highly reactive ROS including hydroxyl radical (OH · −) and peroxynitrite (ONOO−) are generated by ischemia and reperfusion [8, 9]. ROS which induce lipid peroxidation, proteins, and DNA damage in ischemic brain tissue trigger molecular pathways leading to necrosis, apoptosis, and neuroinflammation with subsequent neuronal death and memory and/or motor dysfunction. Therefore, it is important to know the ROS generation in the brain following ischemia/reperfusion in the pathophysiologic process leading to ischemic tissue damage.

To obtain direct evidence of ROS generation in disease pathogenesis, highly sensitive and specific optical probes (fluorescent, luminescent, or chemiluminescent probes) for detecting ROS are being developed [10–13]. However, it is very difficult to directly detect ROS in the brain because they are extremely reactive and their life span is very short. In the brain during or after ischemia, ROS generation has been detected by using electron spin resonance and a microdialysis method [14, 15]. Murakami et al. [16] have reported that hydroethidine can detect the O_2_ · − produced by occlusion of the middle cerebral artery using mutant mice with a heterozygous knock-out gene encoding mitochondrial manganese superoxide dismutase (Mn-SOD). The areas of ROS in both the ischemic core and the peri-infarct area in the permanent and transient middle cerebral artery occlusion (tMCAO) model were detected by using a novel fluorescence probe [17]. Striatal ROS generation in ischemic brain was measured by using salicylate, a hydroxyl radical trapping agent, and a microdialysis method [18].

We have recently reported the usefulness of the radical trapping radiotracer, [^3^H]-labeled *N*-methyl-2,3-diamino-6-phenyl-dihydrophenanthridine ([^3^H]hydromethidine) for detecting ROS generation in the brain [19]. We have already shown that [^3^H]hydromethidine is converted to oxidized products by a superoxide radical (O_2_ · −) and a hydroxyl radical (OH · −). Brain ROS generation induced by cerebral microinjection of sodium nitroprusside could be autoradiographically detected by intravenous administration of [^3^H]hydromethidine. We concluded that [^3^H]hydromethidine rapidly and freely penetrated into the brain where it was rapidly converted to oxidized forms, which were trapped there in response to ROS.

In the present study, we investigated the spatiotemporal changes of ROS generation after ischemia/reperfusion in the tMCAO model mouse using the radical trapping radiotracer [^3^H]hydromethidine. In addition, the effects of 1,3-dimethyl-2-thiourea (DMTU), a hydroxyl radical scavenger, on ROS generation and ischemic damage following tMCAO in mice were also assessed to determine whether its effect might be responsible for hydroxyl radical scavenging action.

## Methods

### Animals and dimethylthiourea (DMTU) administration

**Fig. 1:**
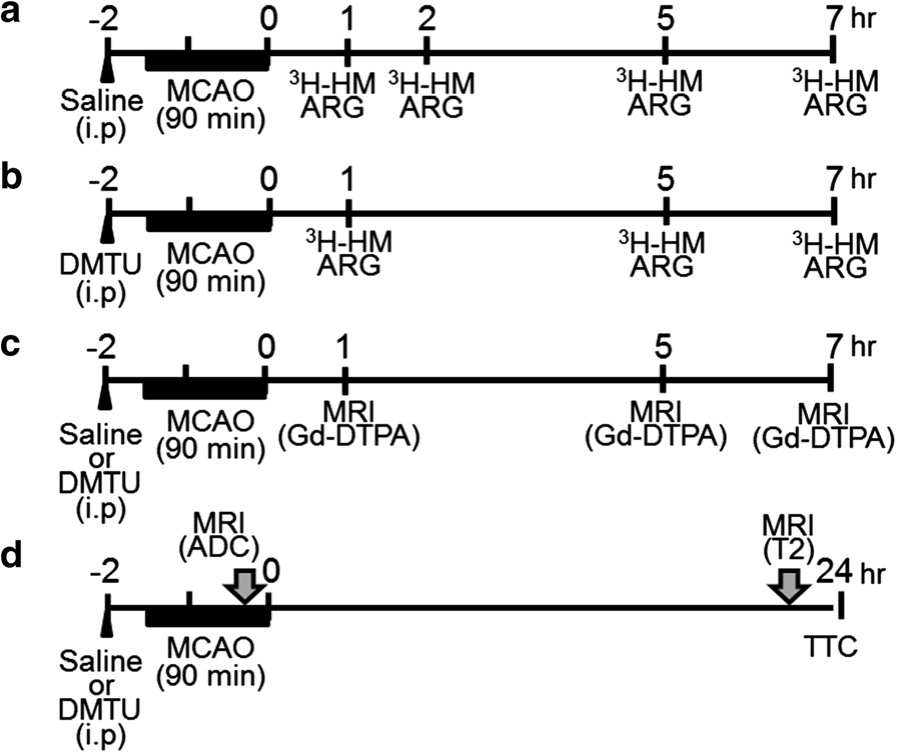
Schematic diagram of the experimental protocol. **a** ROS imaging assessment using autoradiography (*ARG*) with [^3^H]hydromethidine (^3^H-HM) at 1, 2, 5, and 7 h after tMCAO (90 min) in the saline group (*n* = 7–8 in each time point). **b** ROS imaging assessment using ARG using ^3^H-HM at 1, 5, and 7 h after tMCAO (90 min) in the DMTU group (*n* = 5–8 in each time point). **c** BBB permeability assessment using contrast enhanced MRI with Gd-DTPA at 1, 5, and 7 h after tMCAO (90 min) in the saline or DMTU group (*n* = 3–5 at each time point). **d** Cerebral infarction assessment using T2-MRI and TTC staining at 24 h after tMCAO (90 min) in the saline and DMTU groups (*n* = 12–13 in each group). Saline or DMTU was injected 30 min before initiation of MCAO. All MRI experiments were performed under isoflurane anesthesia. *ADC* apparent diffusion coefficient, *T2* T2-weighted MR imaging, *Gd-DTPA* gadolinium diethylenetriaminepentaacetate, *TTC* 2,3,5-triphenyltetrazolium chloride

All animal experiments in the present study were reviewed and approved by the Institutional Animal Care and Use Committee of Shionogi Research Laboratories (Osaka, Japan) and were consistent with the internal guidelines for animal experiments and in adherence to the ethics policy of Shionogi & Co., Ltd (Osaka, Japan). Male C57BL/6 J mice were obtained from CLEA Japan, Inc. (Tokyo, Japan). They were allowed free access to chow and tap water and housed in a temperature-controlled room maintained on a 12-h light/dark cycle with lights on at 8:00 a.m. At 9–11 weeks of age, the mice were randomly assigned to each experiment (Fig. 1a, b, ROS imaging using autoradiography with [^3^H]hydromethidine; Fig. 1c, BBB permeability using contrast-enhanced magnetic resonance imaging (MRI) with gadolinium diethylenetriaminepentaacetate (Gd-DTPA); Fig. 1d, cerebral infarction using T2-MRI and 2,3,5-triphenyltetrazolium chloride (TTC) staining).

DMTU (Sigma-Aldrich, St. Louis, MO, USA), a hydroxyl radical scavenger, was dissolved in saline. Mice were given 200 mg/kg DMTU (20 mg/mL) or saline intraperitoneally 30 min before tMCAO. This dose was selected based on a previous study indicating its neuro protective effect in the in vivo ischemic model [20].

### Transient middle cerebral artery occlusion (tMCAO)

Mice were anesthetized with isoflurane: 5 % induction and 1.5–2 % maintenance. Body temperature was maintained at 37 ± 0.5 °C using a hot plate during surgery. tMCAO was induced using the intraluminal filament technique. Briefly, in the supine position, a nylon 8–0 monofilament suture (Ethicon, Somerville, NJ, USA) coated with silicone resin (Xantopren; Bayer Dental Material, Osaka, Japan) was inserted from the left common carotid artery into the internal carotid artery to occlude the origin of the middle cerebral artery. Reperfusion was performed by withdrawing the suture 90 min after MCAO. Successful occlusion of the middle cerebral artery was verified using MRI (Agilent 7 T/210, Agilent Technologies, CA, USA) because diffusion-weighted images (DWI) and apparent diffusion coefficient (ADC) maps have been established as markers of acute ischemic brain injury. For MRI scans, the animals were subjected to anesthesia using isoflurane (5 % induction, 1.5–2 % maintenance). Mice were placed in a folder for MRI measurement 60 min after MCAO. Respiration rate was monitored by a pneumatic sensor (SA Instruments, Stony Brook, NY, USA). Body temperature was monitored with a rectal fiber-optic probe, and constant warm airflow was utilized to avoid hypothermia. DWI was acquired by EPI sequence. The imaging parameters were TR = 3000 ms, TE = 38.94 ms, shot = 8, matrix size = 0.2 × 0.2 mm^2^, number of averages = 2, number of slices = 6, slice thickness = 0.75 mm, *b* = 0 and 1000 s/mm^2^, and three diffusion directions. ADC map was calculated from DWI using VnmrJ version 4.0 (Agilent Technologies, CA, USA). From DWI images, pixel-by-pixel maps of ADC were estimated. The reduction area in ADC was calculated from six slices based on the threshold value (0.52 × 10–3 mm^2^/s).

### In vivo ROS detection by [^3^H]hydromethidine

[^3^H]Hydromethidine (specific activity: 74 GBq/mmol, radiochemical purity: 98.8 %) was synthesized by *N*-methylation using [methyl-3H]methyl nosylate as described previously [19]. [^3^H]Hydromethidine was diluted with distilled water containing 5 % dimethyl sulfoxide (DMSO) (*v*/*v*), giving 95.7 % radiochemical purity.

**Fig. 2:**
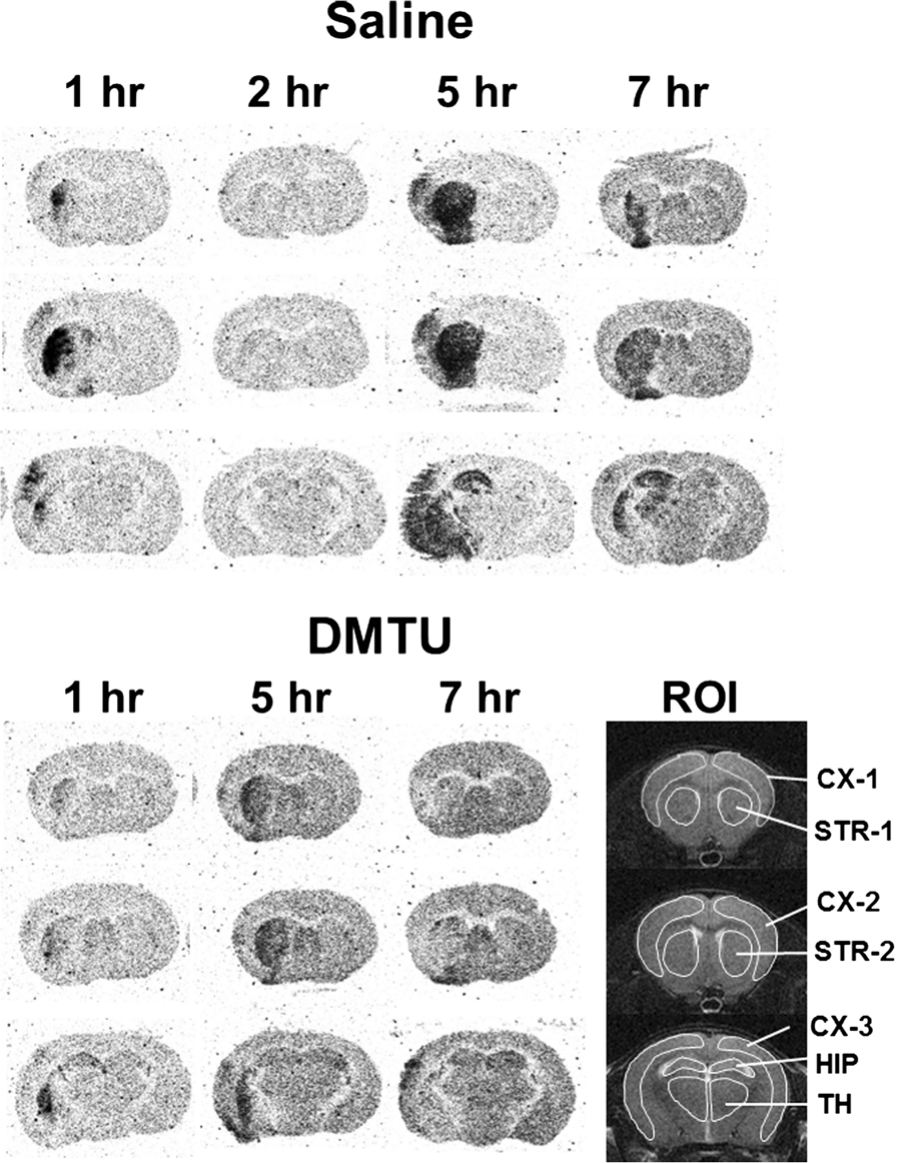
Typical autoradiograms of the brain obtained at 60 min after [^3^H]hydromethidine injection to mice. Typical autoradiograms of the brain after intravenous injection of [^3^H]hydromethidine to mice treated with saline or DMTU (200 mg/kg, i.p.) 30 min before tMCAO (90 min). Coronal sections at the level of the striatum and hippocampus were prepared at 1, 2, 5, and 7 h after ischemia/reperfusion of MCA in mice. Regions of interest (*ROIs*) were marked on the MRI-T2 images in order to quantify the radioactivity concentration

[^3^H]Hydromethidine (740 kBq) was dissolved in an aqueous solution (5 % DMSO, *v*/*v*) and was injected intravenously into the tail vein of mice at 1, 2, 5, and 7 h after tMCAO. The mice were sacrificed by decapitation at 60 min after tracer injection under deep anesthesia with isoflurane. The brains were rapidly removed and frozen, and sections (20 μm thick) were prepared using a cryostat. The sections were exposed to an imaging plate (BAS-TR, GE Healthcare, Tokyo, Japan) for 14 days. After exposure, the plates were read with an FLA-3000 instrument (Fujifilm Corp.). To quantify the radioactivity in the autoradiograph, regions of interest (ROIs) were set as shown Fig. 2, and the photo-stimulated luminescence (PSL) value for each ROI ((PSL − Background)/mm^2^) was determined using Multi Gauge version 2.3 (Fujifilm Corp.) and was calibrated at becquerel per milligram of tissue using the [^3^H]-scale (RPA507, American Radiolabeled Chemicals, Inc., St. Louis, MO, USA).

### Measurement of BBB permeability

To evaluate the BBB permeability, 0.5 mmol/kg of Gd-DTPA (Magnevist, Bayer Schering Pharma, Berlin, Germany) was injected into mice 10 min before MR imaging. T1-weighted images (T1WI) were acquired by spin echo multi-slice sequence before and after injection of Gd-DTPA. The imaging parameters were TR = 500 ms, TE = 10.35 ms, FOV = 25.6 × 25.6 mm, matrix = 96 × 96, slice thickness = 1.0 mm, average = 4, and dummy = 2. After T1WI, T2-weighted images were acquired by spin echo sequence. The imaging parameters were TR = 2500 ms, TE = 50 ms, and average = 1. Image analysis was performed using ImageJ (NIH, Bethesda, MD). Briefly, the percent increase in MRI signal intensity images compared to the respective pixel in the pre-injection images was calculated. To quantify the BBB leakage areas, we set ROIs in two slices shown in Fig. 4a. The threshold was set at the mean percent increase plus two standard deviations in the normal hemisphere. A higher percent increase value than the threshold was considered as BBB disruption areas.

### Measurement of infarct volume

At 24 h after reperfusion of MCAO, the mice were anesthetized by inhalation of 4.0 % isoflurane and killed by decapitation. The brains were quickly removed and chilled in ice-cold saline for 30 s, and 2-mm-thick coronal slices were cut with a tissue slicer. The slices were stained with 2.0 % TTC (Sigma) solution at 37 °C for 10 min and then fixed with 4.0 % phosphate buffered formalin solution. Two sections obtained were scanned with an image scanner and quantified using NIH ImageJ software. Unstained areas (pale colored) were defined as ischemic lesions. The infarct area in each slice was measured using the following formula: ((area of the contralateral hemisphere − area of non-ischemic ipsilateral hemisphere)/area of the whole contralateral hemisphere) × 100. The striatum, cortex, hippocampus, thalamus, and total infarct volumes were determined and presented as a percentage of the volume of the contralateral hemisphere [21].

### Statistical analysis

Data are presented as means ± SD Data were analyzed using analysis of variance (ANOVA) followed by Dunnett’s test for multiple comparisons. Student’s *t* test for two samples was also used. Differences with a value of *P* < 0.05 were considered statistically significant.

## Results

### Brain ROS following tMCAO in mice pretreated with saline or DMTU

**Fig. 3:**
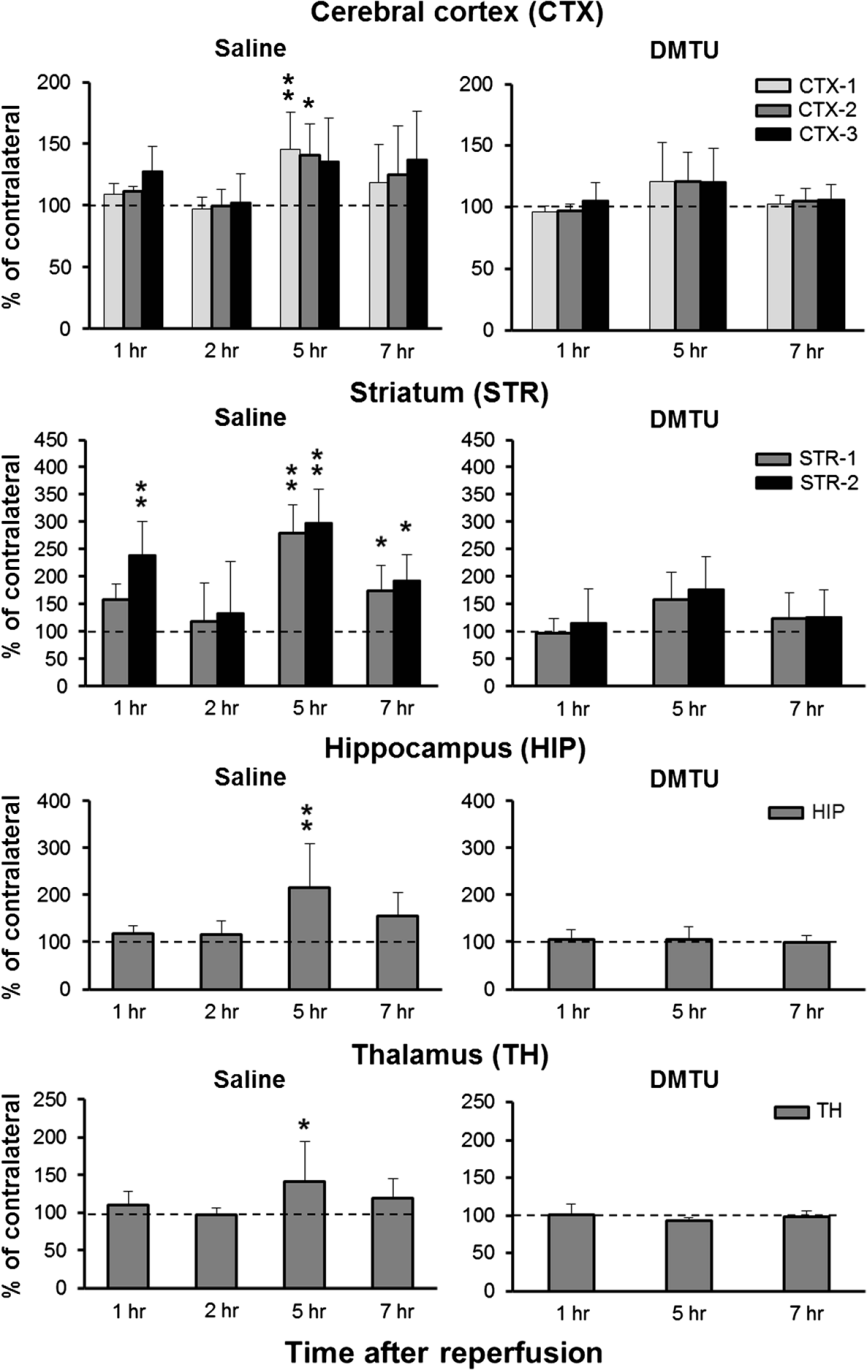
Time changes in brain ROS generation detected by [^3^H]hydromethidine in tMCAO mouse treated with saline and DMTU. [^3^H]Hydromethidine was intravenously injected into mice treated with saline or DMTU (200 mg/kg, i.p.) 30 min before tMCAO (90 min). Coronal sections at the level of the striatum and hippocampus were prepared at 1, 2, 5, and 7 h after ischemia/reperfusion of MCA in mice. Radioactivity concentrations in the brain including the cortex, striatum, hippocampus, and thalamus were calculated from the autoradiograms based on the ROIs drawn on MRI images (Fig. 2) and are presented as the percentage of the contralateral side. Data are expressed as mean ± SD (*n* = 5–8 for each time point). *P < 0.05, **P < 0.01, as compared with control mice

Figure 2 shows typical autoradiograms of the brain obtained at 60 min after [^3^H]hydromethidine injection to mice treated with saline or DMTU (200 mg/kg, intraperitoneally (i.p.)) 30 min before tMCAO. Table 1 and Fig. 3 show the time change of brain ROS generation detected by [^3^H]hydromethidine in tMCAO mouse pretreated with saline or DMTU.

**Table 1:** Radioactivity concentration of the brain after intravenous injection of [^3^H]hydromethidine to tMCAO mice treated with saline and DMTU

Brain region			CTX-1 (Bq/mg)	CTX-2 (Bq/mg)	CTX-3 (Bq/mg)	STR-1 (Bq/mg)	STR-2 (Bq/mg)	HIP (Bq/mg)	TH (Bq/mg)
Control		Right	30.5 ± 2.7	30.7 ± 3.0	29.1 ± 1.3	30.5 ± 2.3	31.2 ± 2.5	30.6 ± 2.7	35.3 ± 3.6
		Left	30.2 ± 2.7	31.0 ± 2.8	29.6 ± 2.5	30.3 ± 2.5	31.2 ± 2.7	30.2 ± 3.7	34.9 ± 3.9
tMCAO + Saline	1 h	Right (C)	25.9 ± 2.0	26.1 ± 1.2	26.3 ± 2.0	26.5 ± 1.4	27.0 ± 1.5	29.9 ± 2.1	29.8 ± 2.5
		Left (I)	28.0 ± 1.6	29.1 ± 1.1	33.4 ± 5.0	41.9 ± 7.2	64.4 ± 17.2	34.8 ± 6.0	32.7 ± 4.6
	2 h	Right (C)	37.9 ± 9.1	37.6 ± 8.9	37.0 ± 8.3	39.1 ± 9.0	39.3 ± 9.5	43.6 ± 12.0	42.2 ± 8.8
		Left (I)	36.4 ± 6.5	36.9 ± 6.8	37.0 ± 7.3	44.9 ± 23.8	50.4 ± 31.4	49.1 ± 9.5	40.7 ± 5.5
	5 h	Right (C)	21.4 ± 2.6	21.3 ± 2.2	20.9 ± 2.4	22.1 ± 2.9	22.7 ± 2.9	23.8 ± 2.8	24.4 ± 2.8^*^
		Left (I)	31.1 ± 7.2	30.0 ± 6.5	28.7 ± 10.0	62.5 ± 18.5^*^	68.4 ± 19.8^*^	49.7 ± 20.2	34.2 ± 12.4
	7 h	Right (C)	36.2 ± 5.5	36.0 ± 5.5	35.6 ± 5.4	37.7 ± 5.8	38.1 ± 5.7	40.3 ± 7.5	41.6 ± 4.6
		Left (I)	42.6 ± 11.0	44.7 ± 14.1^*^	48.3 ± 13.5^**^	64.7 ± 15.5^**^	72.7 ± 18.2^**^	62.2 ± 21.2^**^	49.5 ± 10.6^*^
tMCAO + DMTU	1 h	Right (C)	34.6 ± 7.7	34.7 ± 7.1	33.5 ± 5.8	35.2 ± 6.6	36.1 ± 7.2	41.4 ± 8.3	38.3 ± 7.6
		Left (I)	33.1 ± 6.8	33.5 ± 5.8	35.2 ± 7.4	34.0 ± 9.8	42.8 ± 20.6	43.7 ± 15.1	38.2 ± 6.9
	5 h	Right (C)	38.6 ± 4.9	38.6 ± 4.7	38.1 ± 4.5	40.8 ± 5.9		49.2 ± 7.3^**^	44.3 ± 5.8
		Left (I)	46.4 ± 13.5^*^	46.5 ± 10.0^*^	46.0 ± 11.6^*^	62.5 ± 19.9^*^	69.1 ± 18.8^*^	51.1 ± 12.0	41.3 ± 4.2
	7 h	Right (C)	62.4 ± 10.5^**^	61.6 ± 10.0^**^	61.2 ± 9.5^**^	64.8 ± 11.4^**^	64.4 ± 11.6^**^	78.3 ± 14.0^**^	66.9 ± 10.9^**^
		Left (I)	63.8 ± 10.7^**^	64.3 ± 10.1^**^	64.2 ± 8.3^**^	79.0 ± 19.5^**^	80.6 ± 19.6^**^	76.7 ± 15.1^**^	65.8 ± 11.2^**^

[^3^H]Hydromethidine was intravenously injected into mice treated with saline or DMTU (200 mg/kg, i.p.) 30 min before tMCAO (90 min). Coronal sections at the level of the striatum and hippocampus were prepared at 1, 2, 5, and 7 h after ischemia/reperfusion of MCA in mice. Data are expressed as mean ± SD (Bq/mg, *n* = 5–8 for each time point)

*C* contralateral, *I* ipsilateral

*P < 0.05, **P < 0.01, as compared with control mice

In tMCAO mouse, increased radioactivity was observed in the ipsilateral hemisphere. Radioactivity was seen in the striatum, cortex, and adjacent brain areas. We also found that in some mice, radioactivity accumulation was observed in the hippocampus. The increase in radioactivity indicating ROS generation was first observed in the ipsilateral striatum and cortex 1 h after tMCAO in mice. The increase of radioactivity was attenuated at 2 h after tMCAO, and then, it reached maximum at 5 h. The high accumulation of radioactivity remained until 7 h after tMCAO. In the striatum (STR-2), the radioactivity concentrations of the ipsilateral side to the contralateral side were 239 % at 1 h, 299 % at 5 h, and 192 % at 7 h after tMCAO. DMTU treatment significantly attenuated the accumulation of radioactivity in the ipsilateral hemisphere at 1, 5, and 7 h after tMCAO. In addition, DMTU treatment significantly increased the concentration of radioactivity in the contralateral hemisphere at 7 h after tMCAO (Table 1).

### BBB permeability following tMCAO in mice pretreated with saline or DMTU

**Fig. 4:**
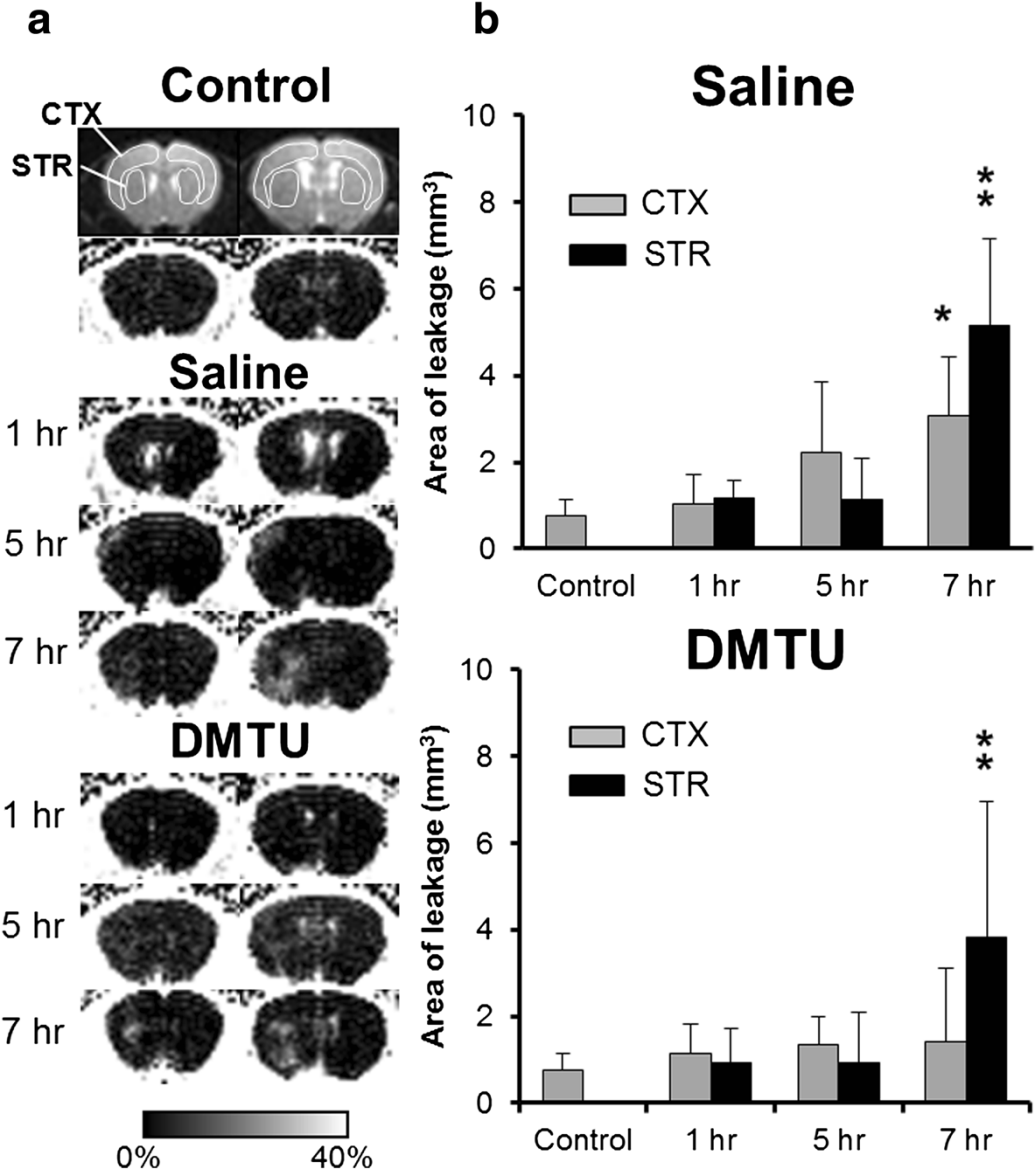
Time changes in BBB permeability in the cortex and striatum following tMCAO in mice treated with saline or DMTU. **a** Representative of Gd-DTPA-enhanced MR images at 1, 5, and 7 h after ischemia/reperfusion in mice treated with saline and DMTU. **b** Area of Gd-DTPA leakage was evaluated as BBB permeability. T1-weighted images (*T1WI*) were acquired by spin echo multi-slice sequence before and after injection of Gd-DTPA. Data are expressed as mean ± SD (*n* = 3–5 for each time point). *P < 0.05, **P < 0.01, as compared with control mice

Figure 4a shows typical contrast-enhanced MR images in tMCAO mice treated with saline or DMTU. As shown Fig. 4, quantitative assessment of Gd-DTPA contrast enhancement in the cortex and striatum (Fig. 4b) showed Gd-DTPA leakage meaning BBB disruption was observed in the ipsilateral cortex and striatum at 7 h after ischemia/reperfusion. At 1 and 5 h, BBB leakage was not clearly observed in the ipsilateral cortex and striatum. In the group of DMTU treatment, the area of leakage was increased in the striatum at 7 h after ischemia/reperfusion. DMTU treatment slightly decreased the area of leakage in the cortex at 7 h after ischemia/reperfusion.

### Effects of DMTU in brain infarction following tMCAO in mice

**Fig. 5:**
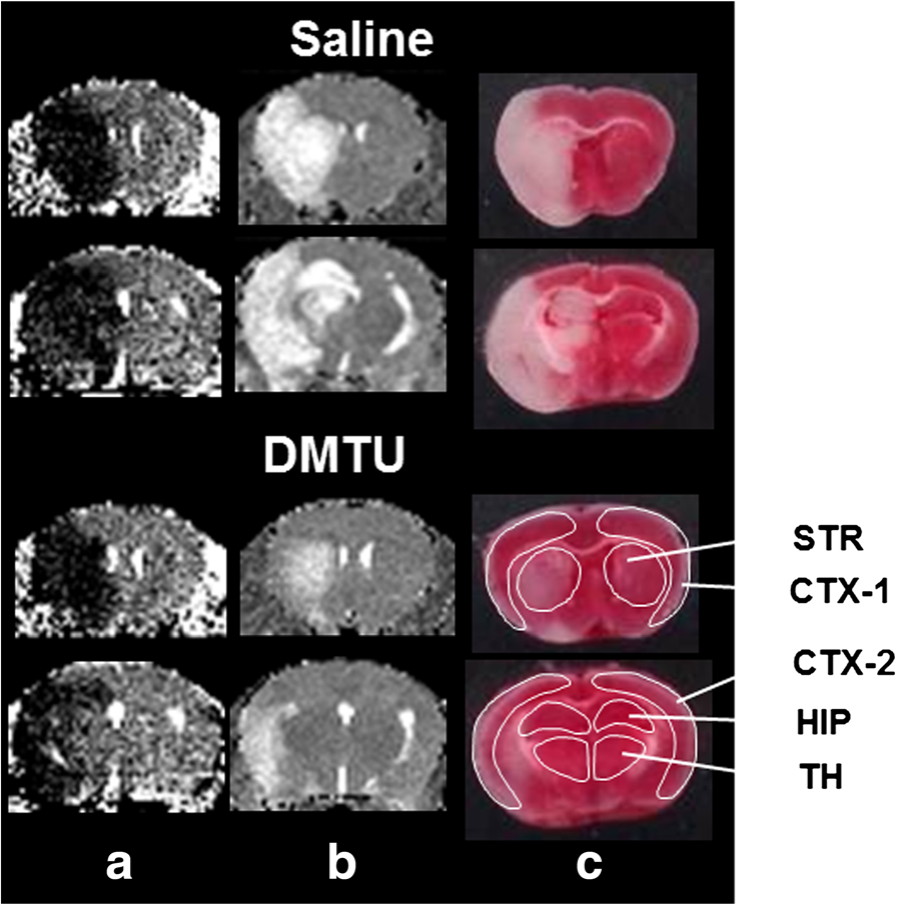
Typical brain images following tMCAO in mice treated with saline or DMTU. **a** ADC map during MCAO indicating acute ischemic damage area in the brain acquired by MRI at 60 min after MCAO. **b** T2WI-weighted images indicating infarct tissues (*white region*) in the brain acquired by MRI at 24 h after ischemia/reperfusion of MCAO. **c** TTC-stained coronal sections (2 mm thick) indicating infarct tissues (*pale unstained region*) at 24 h after ischemia/reperfusion of MCAO. Mice were treated with saline or DMTU 30 min before initiation of MCAO

**Fig. 6:**
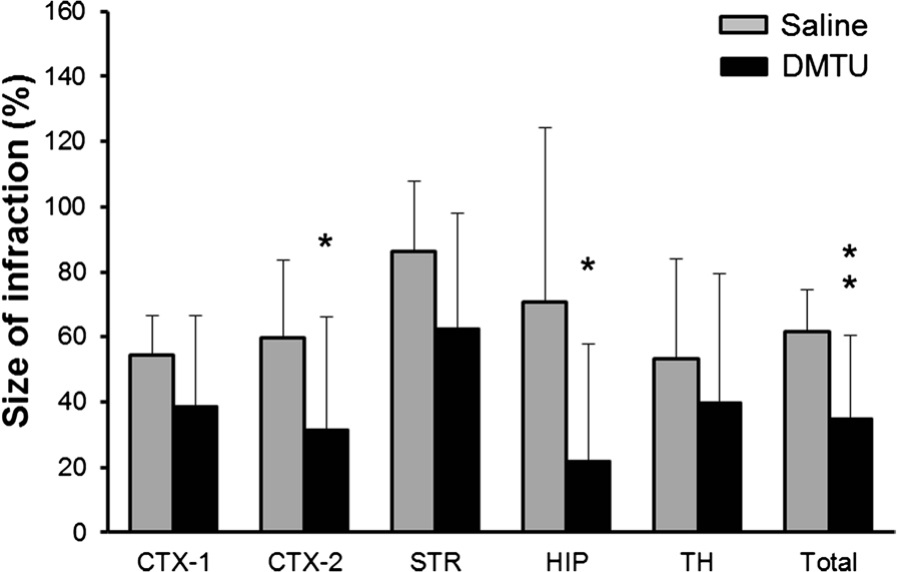
Effect of DMTU on infarct volume following tMCAO in mice. Infarct volume of the total, cortex (*CTX-1*, *CTX-2*), striatum (*STR*), hippocampus (*HIP*), and thalamus (*TH*) of mice treated with vehicle or DMTU (100 mg/kg, i.p.) 30 min before tMCAO (90 min) followed by 24-h reperfusion. The infarct area in each slice was measured by the following formula: ((area of the contralateral hemisphere − area of non-ischemic ipsilateral hemisphere)/area of the whole contralateral hemisphere) × 100. The infarct volume was determined by summing up the infarct area of the 1–2 sections and is presented as the percentage of the volume of the contralateral hemisphere. Data are expressed as mean ± SD (*n* = 12–13 for each group). *P < 0.05, **P < 0.01, as compared with saline

Figure 5 shows typical images of the brain following tMCAO. Figure 5a shows ADC maps at 60 min during ischemia. There was no difference in the reduction area of ADC between the saline- and DMTU-treated mice (56.1 ± 11.9 and 54.7 ± 9.5 mm^3^ in the saline and DMTU groups, respectively; *P* = 0.809), suggesting that acute ischemic damage was not influenced by DMTU treatment. Figure 5b, c shows T2WI-weighted images and TTC-stained sections at 24 h after tMCAO, respectively. The contralateral hemisphere was stained as normal tissue. A part of the cerebral cortex and the striatum in the ipsilateral hemisphere were not stained for mice treated with saline. We also found that in some mice, the thalamus and hippocampus were injured. The T2 hyperintensity area seemed to be comparable with the unstained area of TTC. As shown in Fig. 6, the percentage of the cerebral cortex (CTX-2) and striatum (STR) was 59.6 ± 24.1 % and 86.3 ± 21.6 % in the saline group, respectively. The infarct volume was significantly attenuated by DMTU treatment. The total infarct volumes of the saline and DMTU groups were 61.8 ± 12.7 % and 34.9 ± 25.5 % (*P* < 0.01 compared with saline). Subregional analysis of the cerebral infarct showed that infarcts in the thalamus (TH) and hippocampus (HIP) were 53.2 ± 30.9 % and 70.6 ± 53.5 % in the saline-treated group. The DMTU groups showed a smaller infarct volume in the cortex (31.4 ± 34.9 %) and hippocampus (22.0 ± 35.7 %) (*P* < 0.05 compared with saline).

## Discussion

In the present study, we tried to detect the spatiotemporal change of ROS generation in mouse brain following ischemia/reperfusion. [^3^H]Hydromethidine, a radical trapping radiotracer, was used for ROS detection. In addition, in order to determine whether accumulation of radioactivity after injection of [^3^H]hydromethidine is attenuated by a free radical scavenger in the ischemic region, we investigated the effects of DMTU, a hydroxyl radical scavenger, on ROS generation and brain injury after tMCAO in mice. As shown in Figs. 2 and 3, we found that tMCAO produced a marked increase in ROS production, which peaked at 5 h after reperfusion. Accumulation of radioactivity, indicating ROS generation, was observed in the ipsilateral hemisphere of the tMCAO model mouse. In the striatum, the accumulation was first observed 1 h after reperfusion. The increase of radioactivity was attenuated at 2 h after tMCAO and then reached maximum at 5 h. The accumulation of radioactivity was maintained until 7 h after tMCAO although the level was lower than that at 5 h. The time course of cortical accumulation was also similar to that of the striatum. Hippocampal and thalamic accumulation was also observed at 5 and 7 h after reperfusion in some mice.

Many techniques have been used to detect the generation of ROS in the brain. It is generally detected by indirectly measuring secondary products such as oxidized protein, peroxidized lipids, and oxidized DNA. Direct detection of ROS has been done by using chemiluminescence, fluorescence, spin trapping, and microdialysis [15, 18, 22]. In vivo microdialysis study showed that the formation of 2,3-dihydroxybenzoic acid (2,3-DHBA) and 2,5-dihydroxybenzoic acid (2,5-DHBA), products of salicylate trapping of hydroxyl radicals, increased in the striatum during acute ischemia and reperfusion [18]. Increase of 2,3-DHBA and 2,5-DHBA was observed during ischemia, with high levels being maintained until 2 h after reperfusion.

Temporal and spatial profiles of O_2_ · − after permanent focal ischemia have been examined in mice using in situ staining with dihydroethidium (DHE) [23]. They showed that O_2_ · − generation increased 1 h after MCAO in the ischemic core of the brain and in the boundary area of the infarct zone between 3 and 6 h after permanent focal brain ischemia. Fluorescence imaging using fluorescence probe, 2-[6-(40-hydroxy)phenoxy-3H-xanthen-3-on-9-yl] benzoic acid (HPF) revealed areas of enhanced HPF fluorescence in both the ischemic core and peri-infarct area at 4 h after MCAO in both the permanent and transient MCAO models; the regions generating ROS were more widespread than the areas of ischemic damage [17]. These previous and our findings suggest that ROS generation might be observed at several hours after ischemia/reperfusion. The accumulation of [^3^H]hydromethidine is thought to be due to ROS production because [^3^H]hydromethidine was rapidly converted to oxidized forms, which were trapped there in response to the production of ROS. The amount of trapped oxidized form were mainly dependent upon three factors, the delivery process from plasma (regional cerebral blood flow), the oxidation rate in the brain, and the washout rate of unoxidized [^3^H]hydromethidine from the brain. Therefore, in ischemic brain, the accumulation of [^3^H]hydromethidine might be influenced by cerebral blood flow and BBB permeability. It has been reported to decrease cerebral blood flow in the ischemic side for more than several hours after ischemia/reperfusion [24, 25]. These reports suggest that the accumulation of [^3^H]hydromethidine in the ischemic side was independent of regional cerebral blood flow because regional cerebral blood flow seems to decrease in the ischemic area. On the other hand, BBB disruption has been observed at several hours after ischemia/reperfusion [26, 27]. In the present study, we found that BBB disruption was not observed at 1 or 5 h after ischemia/reperfusion in the tMCAO (90 min) model. These data indicate that the accumulation of [^3^H]hydromethidine at 1 or 5 h after tMCAO might not be affected by BBB disruption. In contrast, the accumulation of [^3^H]hydromethidine at 7 h might be influenced by BBB disruption. However, DMTU reduced the accumulation of [^3^H]hydromethidine although it could not improve striatal BBB disruption at 7 h after ischemia/reperfusion. Thus, our result suggests that the accumulation of [^3^H]hydromethidine in the ischemic area could be due to ROS generation rather than input function such as regional blood flow or BBB disruption. Our interesting finding was that ROS generation was attenuated by the control level at 2 h after reperfusion in the striatum and cortex, ischemic core area. We clearly showed that the accumulation was first observed at 1 h after reperfusion. The increase of radioactivity was attenuated at 2 h after reperfusion, and then, it peaked at 5 h. Antioxidant enzymes, such as superoxide dismutase (SOD) and glutathione peroxidase (GSHP), have been reported to play an important role for ROS generation of tissue affected by ischemia/reperfusion. An increase in GSHP was observed in the parietal cortex at 1 h after ischemia/reperfusion in the tMCAO rat model [28]. These previous findings suggest that ROS generation might be regulated by endogenous antioxidant activity in the brain after ischemia/reperfusion.

ROS such as O_2_ · −, OH · −, and ONOO− are highly reactive molecules that have been implicated in the pathogenesis of secondary neuronal damage after ischemia/reperfusion [29]. The superoxide radical (O_2_ · −) is usually the primary ROS produced and is subsequently converted into hydrogen peroxide (H_2_O_2_) through spontaneous or SOD-catalyzed dismutation. The reaction of O_2_ · − and NO generates the powerful oxidant ONOO−. Reaction of H_2_O_2_ and ONOO− can generate the highly reactive hydroxyl radical (OH · −) [30, 31]. The accumulation of radioactivity observed in the ipsilateral hemisphere suggests that [^3^H]hydromethidine might be oxidized by ROS and then the oxidized form was trapped in the tissue. We recently reported that [^3^H]hydromethidine reacted with O_2_ · − and OH · − but not H_2_O_2_ from the results of an in vitro study. Considering these in vitro results, the accumulation of radioactivity in the brain of our ischemic model strongly suggests that the oxidized form of [^3^H]hydromethidine produced by OH · − or O_2_ · − was trapped in the brain.

We also investigated the effects of DMTU, a hydroxyl radical scavenger, on ROS generation and brain injury after tMCAO in mice. DMTU is often used as a free radical scavenger and can reduce brain damage due to ischemia [32]. It has also been reported that DMTU reduced brain infarction and edema after permanent MCAO in rats [33]. We also showed that DMTU could attenuate brain infarction at 24 h after ischemia/reperfusion in the mouse focal ischemia model. In addition, DMTU attenuated the accumulation of radioactivity in the ipsilateral side of the brain after reperfusion. These results suggest that the accumulation of radioactivity induced by tMCAO is to be mainly due to oxidative conversion of [^3^H]hydromethidine by ROS such as OH · −. On the other hand, DMTU increased radioactivity concentration of [^3^H]hydromethidine in the contralateral side at 7 h after ischemia/reperfusion. The increase of radioactivity concentration might be due to increased cerebral blood flow induced by DMTU treatment. Further studies will be needed to assess the effect of DMTU on cerebral blood flow.

Reactive nitrogen species such as NO and ONOO− play an important role in the process of cerebral ischemia/reperfusion injury [34]. Superoxide can react with NO to produce ONOO− that causes mitochondrial dysfunction, and this leads to brain damage. We recently found that [^3^H]hydromethidine was oxidized by ONOO− in in vitro study (in-house data). Therefore, it is possible that [^3^H]hydromethidine was oxidized by ONOO− in the brain after ischemia/reperfusion. In addition, lipid peroxidation of the cell membrane induced by ROS is considered to oxidize [^3^H]hydromethidine in ischemia/reperfusion in the brain. Thus, it might be difficult to identify the involvement of specific species in brain injury after ischemia/reperfusion because [^3^H]hydromethidine is oxidized by ROS such as O_2_ · −, OH · −, or ONOO−. The present results suggest [^3^H]hydromethidine to be a useful probe for assessing the role of ROS in the ischemic brain state. Time-dependent change of ROS could be detected in the same animals using positron-labeled hydroethidine-related compounds because hydromethidine can be labeled using [^11^C]methylation instead of [^3^H]-labeling. Further studies including small-animal PET studies using [^11^C]hydromethidine should be very useful for studying the pathophysiological roles of ROS in diseases such as ischemic stroke.

## Conclusions

The present study showed that [^3^H]hydromethidine is a useful radiotracer for detecting in vivo brain ROS production after ischemic injury. To the best of our knowledge, this is the first report of the detection of brain ROS generation after ischemia/reperfusion by using a radical trapping radiotracer.
